# Pancreatic acinar cell carcinoma demonstrates marked clinical heterogeneity and recurrent actionable genomic alterations

**DOI:** 10.3389/fcell.2026.1913398

**Published:** 2026-10-13

**Authors:** Noor Farhoud, Raed Al-Rajabi, Joaquina Baranda, Haoran Li, Ameer Hamza, Wei Zhang, Ravi Paluri, Ashish Manne, Prasad Dandawate, Subhrajit Saha, Timothy Schmitt, Sean Kumer, Clay King, Mojtaba Olyaee, Amit Rastogi, Reza Hejazi, Weijing Sun, Anup Kasi

**Affiliations:** 1 Department of Internal Medicine, University of Kansas Medical Center, Kansas City, KS, United States; 2 Department of Internal Medicine, Division of Medical Oncology, University of Kansas Medical Center, Westwood, KS, United States; 3 Department of Pathology and Laboratory Medicine, University of Kansas Medical Center, Kansas City, KS, United States; 4 Department of Hematology and Oncology, Wake Forest School of Medicine, Winston-Salem, NC, United States; 5 Department of Internal Medicine, Division of Medical Oncology, The Ohio State University Comprehensive Cancer Center, Columbus, OH, United States; 6 Department of Cancer Biology, University of Kansas Medical Center, Kansas City, KS, United States; 7 Department of Surgery, University of Kansas Medical Center, Kansas City, KS, United States; 8 Division of Gastroenterology, Department of Internal Medicine, University of Kansas Medical Center, Kansas City, KS, United States; 9 Division of Gastroenterology and Hepatology, University of Kansas Medical Center, Kansas City, KS, United States

**Keywords:** genomic profiling, KRAS wild-type, molecular profiling, PacC, pancreatic acinar cell carcinoma, precision oncology, rare pancreatic cancer, targeted therapy

## Abstract

**Background:**

Pancreatic acinar cell carcinoma (PACC) is a rare pancreatic malignancy with a molecular profile distinct from pancreatic ductal adenocarcinoma (PDAC) but is commonly treated using extrapolated paradigms.

**Methods:**

We retrospectively identified patients with histologically confirmed PACC at a tertiary academic center and abstracted clinical, pathologic, treatment, and outcome data. Next-generation sequencing (NGS) was reviewed for microsatellite status, tumor mutational burden, and actionable genomic alterations.

**Results:**

Seven patients were identified (median age, 64 years; 57% male). Most presented with metastatic disease (71%), yet outcomes were heterogeneous, including prolonged progression-free survival exceeding 7 years and 16 years in select patients. Carbohydrate antigen 19–9 (CA 19–9) was normal in all cases, and jaundice was absent despite frequent pancreatic head involvement. All tumors were KRAS wild-type. NGS revealed recurrent alterations including CDKN2A/B and MTAP co-deletion, BRAF fusion, IKZF1 loss, and BAP1 mutation. Tumors were microsatellite stable with low-to-intermediate tumor mutational burden. One patient received matched targeted therapy through a precision oncology trial.

**Conclusion:**

PACC demonstrates substantial clinical heterogeneity with a subset of patients exhibiting prolonged survival despite metastatic disease. Recurrent actionable genomic alterations support routine molecular profiling and prioritization for biomarker-driven trials. These findings challenge extrapolation from PDAC and support a precision oncology framework for this ultra-rare malignancy.

## 1 Introduction

Pancreatic acinar cell carcinoma (PACC) is an ultra-rare exocrine pancreatic malignancy, accounting for approximately 1% of pancreatic neoplasms (Ikezaw et al., 2024). Unlike pancreatic ductal adenocarcinoma (PDAC), which is characterized by *KRAS* mutations and a highly aggressive clinical course, PACC demonstrates distinct molecular, pathologic, and clinical features (Sakakida et al., 2023; Jiao et al., 2014; Chmielecki et al., 2014). Despite these differences, management of PACC is commonly extrapolated from PDAC, and optimal treatment strategies remain poorly defined.

In this single-center cohort, we characterize the clinicopathologic and genomic features of PACC. Patients frequently lacked typical markers of pancreatic malignancy, including elevated CA 19–9 and obstructive jaundice. A high proportion presented with metastatic disease; however, outcomes were variable, with some patients demonstrating prolonged survival despite advanced stage. Molecular profiling revealed recurrent, potentially actionable alterations, supporting the use of genomic testing. These findings highlight the diagnostic challenges and therapeutic opportunities in PACC.

## 2 Materials and methods

A retrospective review of histologically confirmed PACC at a single tertiary academic center was performed. Institutional pathology database and electronic medical records were used to identify patients. Those with mixed histology were included if acinar differentiation constituted the predominant component on pathology. Clinical, radiographic, pathologic, treatment, and outcome data were extracted. Standard-of-care NGS results were reviewed, with focus on potentially actionable alterations. This retrospective study was reviewed by the Institutional Review Board and determined to be exempt (Study ID: STUDY00161040). The study utilized existing clinical, demographic, and molecular data obtained as part of routine patient care. No additional patient contact, intervention, or genetic testing was performed for research purposes. Next-generation sequencing was performed as part of routine standard-of-care testing using commercially available platforms (Caris Molecular Intelligence, Tempus xT, Guardant360, and Ambry Genetics), which varied in gene content across patients and included detection of single-nucleotide variants, insertions/deletions, copy number alterations, and gene fusions. Testing was not uniform across the cohort.

## 3 Results

### 3.1 Patient and clinical characteristics

A total of seven patients were identified. The median age at diagnosis was 64 years (range, 46–70), with a slight male predominance (57%). The most common presenting symptoms included abdominal pain, nausea, vomiting, fatigue, changes in appetite, constipation, and diarrhea. Less frequently reported symptoms included pruritus, flank pain, and hematuria. Two patients were asymptomatic on presentation: one was found to have an elevated alkaline phosphatase level prompting further evaluation, and the other had an incidental pancreatic mass detected during imaging performed as part of a kidney transplant work-up. Notably, jaundice was absent. The average duration of symptoms prior to diagnosis was approximately 3 months.

### 3.2 Laboratory and radiologic findings

Among patients with available laboratory data, lipase was elevated in only one case. Albumin and CA 19–9 levels were within normal limits at diagnosis across all patients. Total bilirubin was elevated in two patients. Radiologically, the mean tumor size was 3.73 cm. The majority of tumors were located in the head of the pancreas, with only one arising in the tail. Necrosis was identified radiologically in a single case. Vascular involvement was observed in two patients, both involving the portal vein, and three patients demonstrated regional lymphadenopathy.

### 3.3 Disease extent and histopathology

At presentation, five patients had radiographic evidence of metastatic disease. The liver was the most common site of metastasis (n = 4), while one patient had nodal metastasis. Among those with hepatic involvement, one patient also had concurrent lung metastases and subsequently developed peritoneal spread. Only two patients had resectable disease at the time of diagnosis.

Histopathologic evaluation revealed acinar cell carcinoma in six cases and a mixed acinar–ductal carcinoma in one case. Among the six pure acinar cell carcinomas, one demonstrated focal neuroendocrine differentiation on immunohistochemistry (focal synaptophysin positivity, chromogranin negative) without a true mixed acinar-neuroendocrine carcinoma phenotype. The mixed case showed a ductal component supported by pCEA and mucicarmine positivity, rather than acinar-squamous or acinar-neuroendocrine differentiation. All tumors were high grade. Perineural and lymphovascular invasion were identified in only one patient.

Immunohistochemical analysis demonstrated consistent expression of exocrine markers, with frequent positivity for trypsin and chymotrypsin, and BCL10 expression observed in multiple cases, supporting acinar differentiation. Neuroendocrine markers showed limited and often focal expression. Synaptophysin was occasionally focally positive, whereas chromogranin expression was variable, arguing against a true neuroendocrine phenotype. CD56 expression was also variable, while CD10 was uniformly negative. Histochemical staining revealed periodic acid–Schiff (PAS) positivity in several cases. Polyclonal carcinoembryonic antigen (pCEA) and mucicarmine stains were variably positive. The Ki-67 proliferation index ranged from 20% to 60%, with several tumors demonstrating high proliferative activity (≥50%), consistent with aggressive biologic behavior. Amylase immunostaining was not performed in this cohort. Acinar differentiation was instead established using trypsin, chymotrypsin, BCL10, and PAS positivity, consistent with WHO diagnostic criteria for PACC. Representative H&E images are shown in Figure 2, and representative IHC images are shown in Figure 3.

**FIGURE 1:**
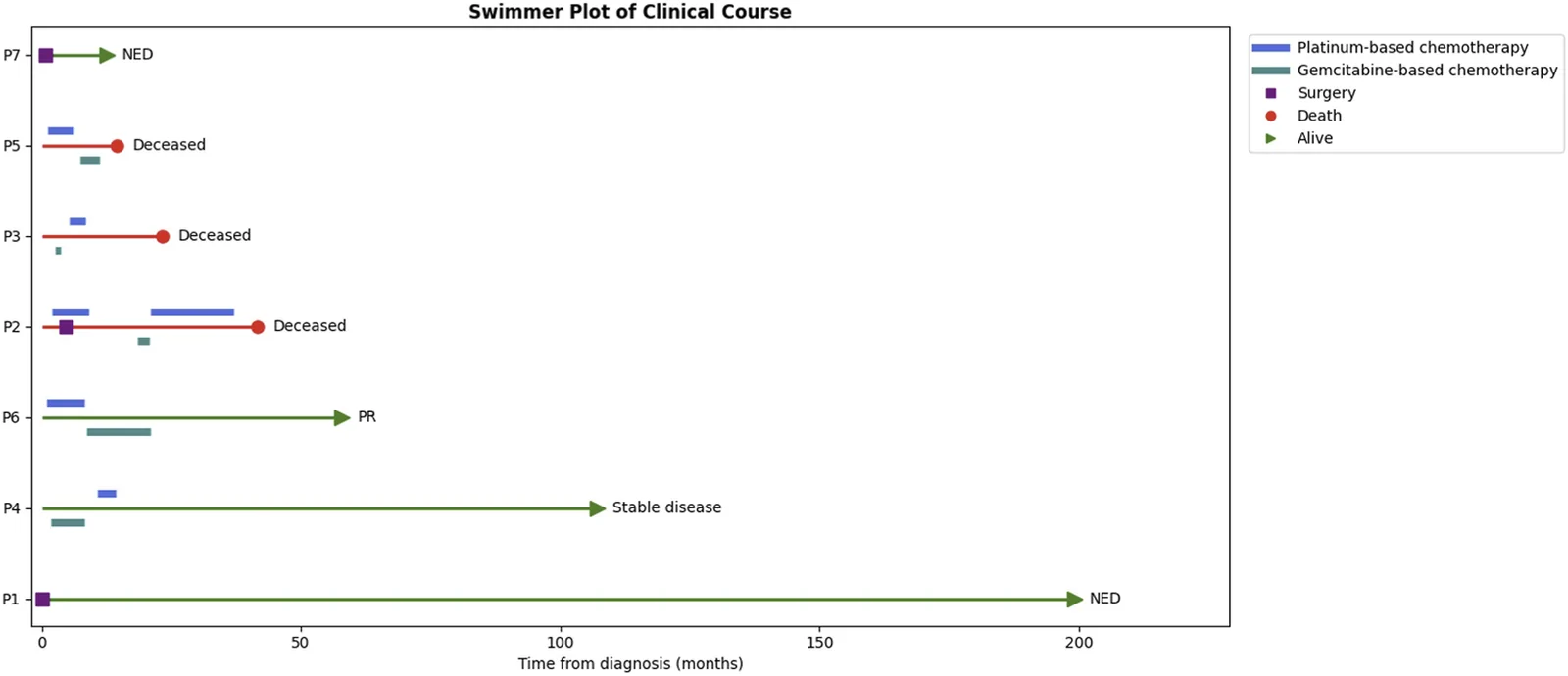
Swimmer plot of clinical course in patients with pancreatic acinar cell carcinoma. Swimmer plot illustrating treatment course, response, and survival outcomes for each patient (P1–P7). Blue bars represent platinum-based therapy; teal bars represent gemcitabine-based therapy. Purple squares indicate surgical resection. Red circles denote death, and green arrows indicate patients alive at last follow-up. Clinical responses are labeled as no evidence of disease (NED) or partial response (PR) where applicable. Time is displayed in months from diagnosis. Abbreviations: NED, no evidence of disease; PR, partial response.

**FIGURE 2:**
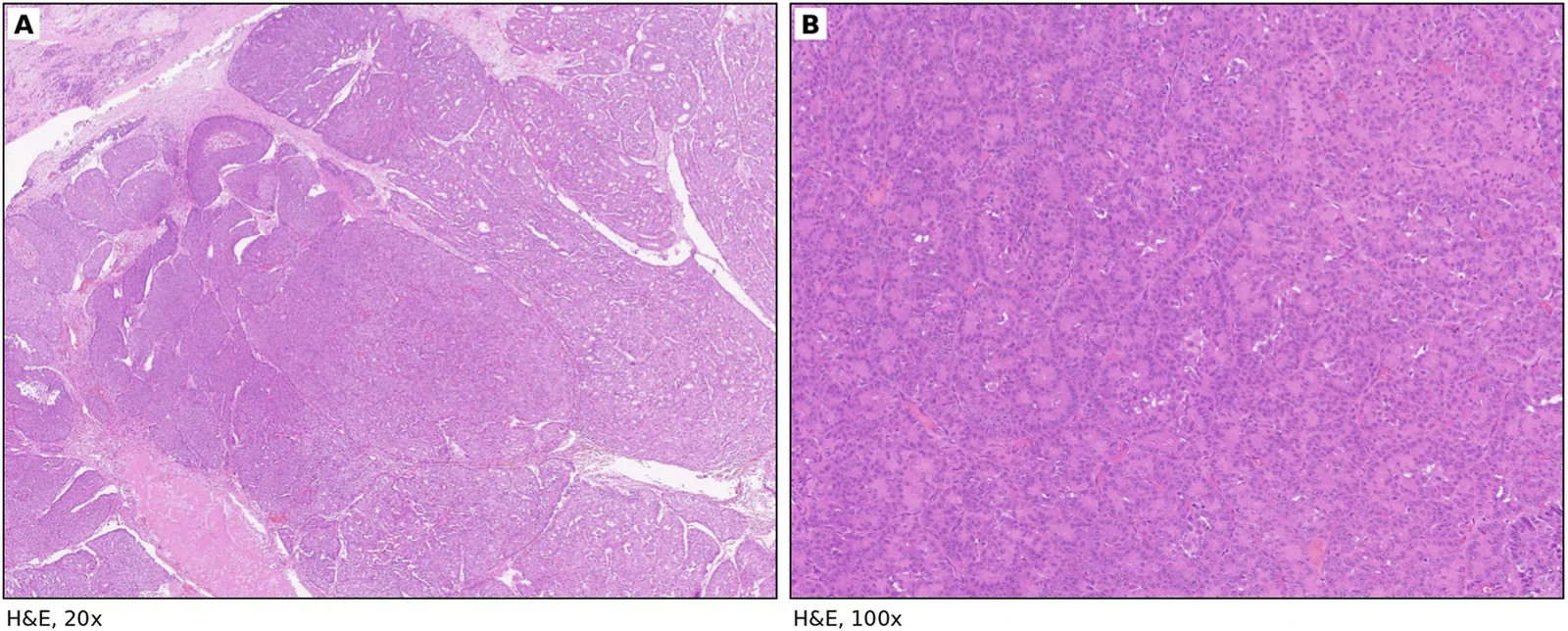
Representative hematoxylin and eosin (H&E) staining of pancreatic acinar cell carcinoma. **(A)** Low-power photomicrograph (H&E, 20×) demonstrating densely cellular acinar and glandular patterns with minimal stroma. **(B)** Medium-power photomicrograph (H&E, 100×) demonstrating cytologic detail, including uniform nuclei with a central prominent nucleolus and eosinophilic cytoplasm.

**FIGURE 3:**
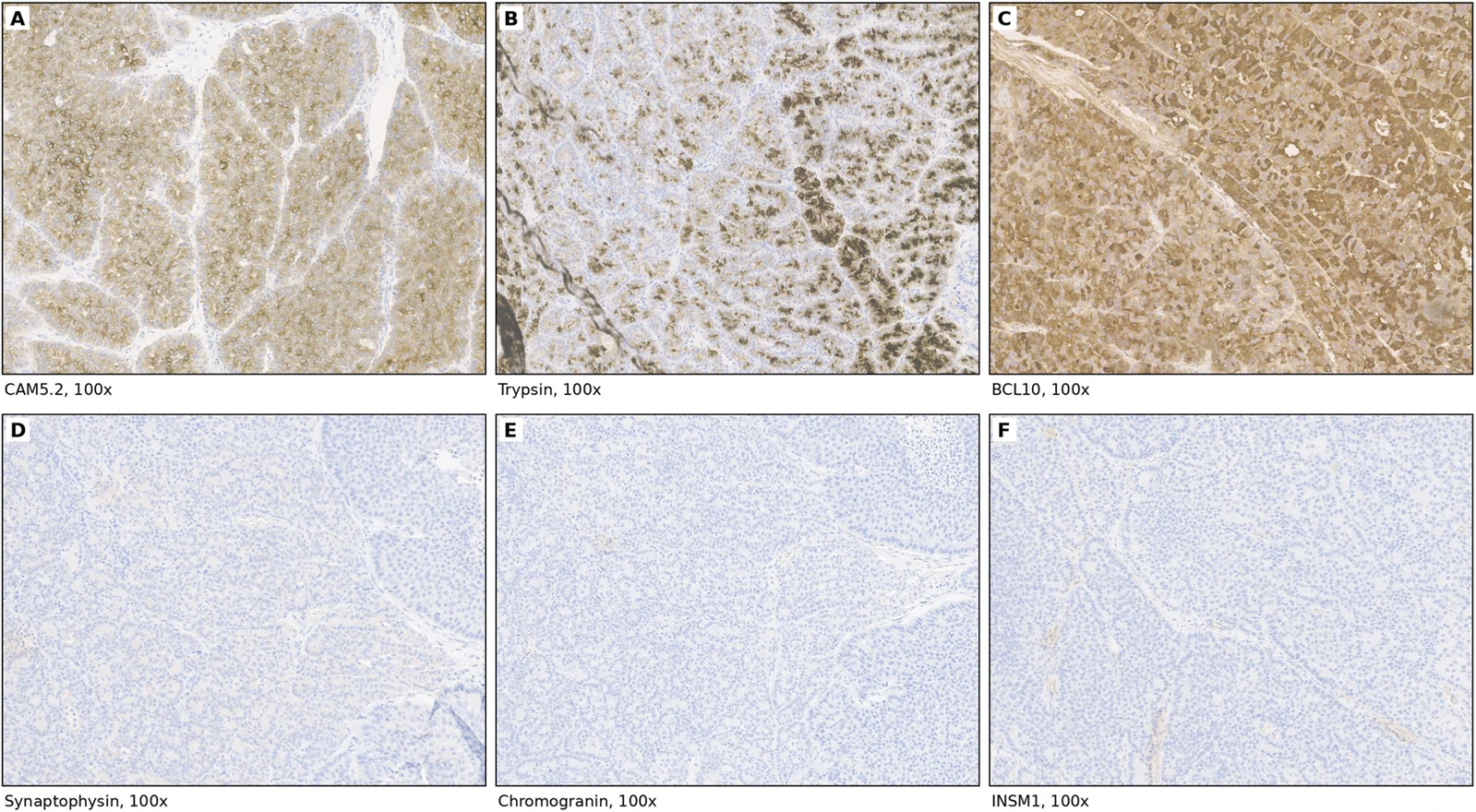
Representative immunohistochemical (IHC) staining of pancreatic acinar cell carcinoma. Tumor cells are immunoreactive for CAM5.2 **(A)**, trypsin **(B)**, and BCL10 **(C)**, and are negative for synaptophysin **(D)**, chromogranin **(E)**, and INSM1 **(F)**, all at ×100 magnification, supporting acinar differentiation without a significant neuroendocrine component.

### 3.4 Molecular profiling

NGS was performed in a subset of cases. Among tested tumors, the majority were microsatellite stable (MSS) with low-to-intermediate tumor mutational burden (TMB ∼6–6.8 mutations/Mb), and no germline alterations were identified. All tumors were *KRAS* wild-type. Actionable alterations included 9p21.3 co-deletion (*CDKN2A/B* and *MTAP* loss), *IKZF1* loss, *SEC24D-BRAF* fusion, and *BAP1* mutation (Table 1). Variants of uncertain significance (VUS) were also detected. Importantly, one case demonstrated a confirmed *BRAF* mutation, prompting consideration for targeted therapy and clinical trial enrollment (NCI-MATCH). NGS was performed in three of seven patients (43%) using different commercial platforms depending on treating institution and testing period. Caris Molecular Intelligence, Tempus xT, and Guardant360 in one patient; Ambry Genetics in one patient; and Ambry Genetics with Tempus xT in one patient. The *CDKN2A/B* and *MTAP* co-deletion was identified by NGS-based copy number analysis. Zygosity (homozygous versus heterozygous) was not specified in the available reports, precluding definitive assessment of eligibility for MTAP-directed therapies.

**TABLE 1:** Clinical, pathologic, and genomic characteristics of patients with pancreatic acinar cell carcinoma (PACC) in a single-center cohort.

Patient	Age	Sex	Stage	Tumor Location	Resection	NGS findings	MSI status	TMB	Potential target
1	46	F	Stage IA	Head	Yes	Not tested	N/A	N/A	N/A
2	68	F	Stage III	Head	Yes	TP53 mutation; KRAS wild-type; CDKN2A/B loss; MTAP loss; SEC24D-BRAF fusion	MSS	Intermediate	BRAF/MEK/PRMT5 inhibitors
3	67	M	Stage IV	Head	Attempted (aborted)	Not tested	N/A	N/A	N/A
4	70	M	Stage IV	Head	No	Not tested	N/A	N/A	N/A
5	86	F	Stage IV	Head	No	BAP1 mutation	MSS	6.6	EZH2 inhibitors
6	55	M	Stage IV	Tail	No	CDKN2A/B loss; MTAP loss; IKZF1 loss; KRAS wild-type	MSS	6.8	PRMT5 inhibitors
7	57	M	Stage IB	Head	Yes	Not tested	N/A	N/A	N/A

Abbreviations: F, female; M, male; NGS, next-generation sequencing; MSI, microsatellite instability; MSS, microsatellite stable; TMB, tumor mutational burden; N/A, not applicable; KRAS, kirsten rat sarcoma viral oncogene homolog; TP53, tumor protein p53; CDKN2A/B, cyclin-dependent kinase inhibitor 2A/2B; MTAP, methylthioadenosine phosphorylase; BRAF, B-Raf proto-oncogene; IKZF1, IKAROS, family zinc finger 1; BAP1, BRCA1-associated protein 1; EZH2, enhancer of zeste homolog 2; PRMT5, protein arginine methyltransferase 5; MEK, mitogen-activated protein kinase kinase.

### 3.5 Treatment and outcomes

Surgical resection was performed in three of seven patients, with one patient receiving neoadjuvant therapy. Postoperative complications were limited to anastomotic leak in two patients. Among those who underwent resection, outcomes were favorable, with two patients remaining alive without evidence of disease at last follow-up.

The four patients who did not undergo surgical resection experienced variable outcomes. Swimmer plot analysis demonstrated that two patients achieved prolonged progression-free survival (>7 years and >16 years), whereas the remaining patients had follow-up durations of less than 5 years (Figure 1). Two patients died of disease despite systemic therapy.

Systemic therapy was administered in the majority of patients (5 of 7), most commonly including platinum- and gemcitabine-based regimens. One patient received targeted therapy with ulixertinib in the NCI-MATCH clinical trial.

Overall survival (OS) was calculated from the date of diagnosis to the date of death or last follow-up. Among the cohort, three patients died during the study period. The observed survival times for these patients were approximately 41 months (Patient 2), 23.5 months (Patient 3), and 14.6 months (Patient 5). The median OS was 23.5 months.

## 4 Discussion

In this series, we further define PACC as molecularly distinct from PDAC. Approximately 70% of patients presented with metastatic disease, exceeding rates reported in prior studies (Chaudhary, 2014; Al-Hader et al., 2017). However, clinical trajectories ranged from rapid progression to prolonged progression-free survival exceeding 90 and 200 months, an uncommon finding in PACC. This discordance challenges the conventional belief that metastatic PACC uniformly leads to a poor prognosis and suggests the presence of distinct subsets, including treatment-responsive disease even in advanced stages.

Molecularly, all tumors were *KRAS* wild-type, reinforcing the distinction between PACC and PDAC, in which *KRAS* mutations predominate (Sakakida et al., 2023; Jiao et al., 2014; Chmielecki et al., 2014). This is therapeutically relevant as it discourages the extrapolation of PDAC-based treatment models to PACC and supports a shift toward genomically driven treatment strategies tailored to the molecular features of PACC.

Clinically, conventional markers of pancreatic malignancy were unreliable. All patients had normal CA 19–9 levels, lipase elevation was rare, and jaundice was absent despite a predominance of pancreatic head tumors. This pattern, consistent with prior reports, likely reflects the expansile growth characteristics of PACC (Chaudhary, 2014). As a result, reliance on traditional biomarkers may delay diagnosis, emphasizing the need for increased clinical suspicion and alternative diagnostic approaches.

Pathologically, tumors demonstrated consistent acinar differentiation, with expression of trypsin, chymotrypsin, and BCL10, alongside variable neuroendocrine marker expression without a true neuroendocrine phenotype. All tumors were high grade. However, perineural and lymphovascular invasion were infrequent, suggesting PACC may preferentially disseminate via early hematogenous spread rather than stepwise local invasion, potentially explaining the high prevalence of metastatic disease at diagnosis.

Molecular profiling revealed genomically actionable alterations despite low tumor mutational burden and microsatellite stability. Recurrent alterations included *BRAF* fusion, *CDKN2A/B* and *MTAP* co-deletion, and *BAP1* mutation, supporting the use of genomic profiling. The *CDKN2A/B*-*MTAP* co-deletion is a frequent focal deletion event across multiple tumor types, including glioblastoma, PDAC, mesothelioma, melanoma, and bladder cancer, and its presence in PACC should not be interpreted as a tumor-specific feature without evidence of relative enrichment. Molecular testing was performed in only three of seven patients in this cohort, limiting the generalizability of these genomic findings to the broader PACC population. One patient received targeted therapy through a precision oncology trial. However, broader clinical implementation remains limited by variant classification, access to targeted therapies, and the rarity of PACC-specific trials. These findings support the need to prioritize PACC patients for biomarker-driven and histology-agnostic clinical trials.

From a therapeutic standpoint, surgical resection remains the cornerstone of curative-intent management and has been associated with improved survival, with prior studies reporting median OS of approximately 30–60 months (Al-Hader et al., 2017). However, recurrence following resection highlights the likelihood of occult micrometastatic disease and warrants consideration of perioperative systemic strategies (Holen et al., 2002). In our cohort, one patient experienced recurrence despite multimodal therapy and ultimately died of disease, emphasizing the aggressive potential of some tumors.

Among patients with unresectable disease, outcomes were variable, and responses to platinum- and gemcitabine-based regimens were inconsistent. Although platinum-based regimens are often favored, existing data remain inconclusive and are largely derived from small cohorts (Kruger et al., 2016; Sridharan et al., 2021; Takahashi et al., 2021; Yoo et al., 2017; Shin et al., 2021). Our findings similarly demonstrate inconsistent benefit, emphasizing the inadequacy of PDAC-based treatment approaches in this population.

This study is limited by its retrospective design and single-center cohort. With only seven patients, these findings should be considered hypothesis-generating rather than confirmatory and are not intended to establish PACC-specific rates of any individual genomic alteration. Nevertheless, it provides detailed clinical annotation and molecular profiling in an ultra-rare malignancy where prospective, biomarker-driven data remain limited. PACC is a molecularly distinct malignancy characterized by the absence of reliable diagnostic biomarkers and the presence of actionable genomic alterations. Metastatic disease should not be uniformly considered rapidly fatal, and outcomes may vary substantially based on underlying tumor biology. These findings support the routine integration of genomic profiling early in the disease course and prioritization of PACC patients for precision oncology trials.

## Data Availability

The datasets generated and analyzed during the current study are not publicly available due to patient privacy and institutional restrictions on the sharing of clinical data but may be available from the corresponding author upon reasonable request and with appropriate institutional approval.
